# Public Opinions Toward Diseases: Infodemiological Study on News Media Data

**DOI:** 10.2196/10047

**Published:** 2018-05-08

**Authors:** Ming Huang, Omar ElTayeby, Maryam Zolnoori, Lixia Yao

**Affiliations:** ^1^ Department of Health Sciences Research Mayo Clinic Rochester, MN United States; ^2^ Department of Computer Science University of North Carolina at Charlotte Charlotte, NC United States

**Keywords:** news, Reuters, public policy, text mining, sentiment analysis, topic modeling, unmet medical need, research priority

## Abstract

**Background:**

Society always has limited resources to expend on health care, or anything else. What are the unmet medical needs? How do we allocate limited resources to maximize the health and welfare of the people? These challenging questions might be re-examined systematically within an infodemiological frame on a much larger scale, leveraging the latest advancement in information technology and data science.

**Objective:**

We expanded our previous work by investigating news media data to reveal the coverage of different diseases and medical conditions, together with their sentiments and topics in news articles over three decades. We were motivated to do so since news media plays a significant role in politics and affects the public policy making.

**Methods:**

We analyzed over 3.5 million archive news articles from Reuters media during the periods of 1996/1997, 2008 and 2016, using summary statistics, sentiment analysis, and topic modeling. Summary statistics illustrated the coverage of various diseases and medical conditions during the last 3 decades. Sentiment analysis and topic modeling helped us automatically detect the sentiments of news articles (ie, positive versus negative) and topics (ie, a series of keywords) associated with each disease over time.

**Results:**

The percentages of news articles mentioning diseases and medical conditions were 0.44%, 0.57% and 0.81% in the three time periods, suggesting that news media or the public has gradually increased its interests in medicine since 1996. Certain diseases such as other malignant neoplasm (34%), other infectious diseases (20%), and influenza (11%) represented the most covered diseases. Two hundred and twenty-six diseases and medical conditions (97.8%) were found to have neutral or negative sentiments in the news articles. Using topic modeling, we identified meaningful topics on these diseases and medical conditions. For instance, the smoking theme appeared in the news articles on other malignant neoplasm only during 1996/1997. The topic phrases HIV and Zika virus were linked to other infectious diseases during 1996/1997 and 2016, respectively.

**Conclusions:**

The multi-dimensional analysis of news media data allows the discovery of focus, sentiments and topics of news media in terms of diseases and medical conditions. These infodemiological discoveries could shed light on unmet medical needs and research priorities for future and provide guidance for the decision making in public policy.

## Introduction

Advances in biomedical research have greatly improved public health and human longevity; however, many biomedical problems remain unsolved, and many diseases still need feasible treatments or cures. Limited societal resources, such as funding and scientific research, are available for health care or anything else. Allocating resources and prioritizing scientific research to maximize health and welfare are important and challenging problems. Systematic investigations of allocation problems would help address unmet medical needs and promote public health. Appropriate understanding of resource allocation mechanisms in health care would also greatly benefit governmental and private funding agencies, biotechnology and pharmaceutical companies, and scientists and clinicians.

Scientists have gradually paid more attention to the notable problems of resource allocation and research prioritization in health care and have explored different methods to better understand resource allocation mechanisms. Measurement of resource allocation typically requires determination of disease burden and its association with available resources. Measures of disease burden are often quality-adjusted life years (QALYs), disability-adjusted life years (DALYs), or other health-related quality-of-life metrics. In 1999 and 2011, Gross et al [1] and Gillum et al [2] investigated the relationship between National Institutes of Health (NIH) funding and disease burden by studying the number of deaths, disease prevalence and incidence, and length of hospital stay for 29 common diseases and medical conditions. Both studies indicated a correlation between NIH funding and disease burden as calculated with DALYs and suggested that some diseases are overfunded (such as HIV, diabetes, and breast cancer) and some are underfunded (for example, depression and injuries). Sampat et al [3] investigated 107 diseases and medical conditions and determined a strong correlation between clinical trial funding from NIH and deaths or length of hospital stay. These studies examined the resource allocation problem by relating 1 or 2 resource factors to disease burden measured by DALYs and others. The practical calculation of DALYs or QALYs is usually expensive, since it requires surveying the preferences of the target population [4,5]. These works were hindered by data scarcity and examined up to 107 common diseases at arbitrary single time points, which limits the rigorousness and transferability of these studies.

In addition, scientists have proposed using conventional survey methods to include the opinions of citizens, patients, and experts when defining unmet medical needs and research priorities. For example, Dowsett et al [6] interviewed 420 oncologists about the most important research questions in breast oncology. Annals of Oncology launched a new section called Research Needs, which reports on 10 to 15 high-priority research topics. The list of high-priority topics is selected and discussed among biomedical experts using the Delphi method. Cardoso et al [7] published the first article in Annals of Oncology that used this strategy to report research needs in breast cancer. However, the conventional survey method is qualitative and does not efficiently or systematically detect unmet health needs.

In 2015, we introduced the research opportunity index (ROI) and public health index (PHI) for gauging resource allocation based on the assumption that the resources should be proportionally aligned with the burden of each disease [8]. ROI quantitatively measures resource allocation for a particular disease, and PHI assesses resource allocation for all medical conditions as a whole by considering public health needs (as measured by disease prevalence and financial cost), biomedical research (as calculated by the number of published articles), and clinical developments (as determined by the number of clinical trials). These quantitative indices are very flexible and can integrate many other quantifiable factors for research prioritization and resource allocation in biomedicine. Our analysis showed that resource allocation is influenced by previous research and that the current resource allocations are far greater than health needs, thereby resulting in a massive imbalance between research and development investments and US health needs.

More recently, we applied an infodemiological approach [9] to investigate if disease burden for thousands of diseases and medical conditions can be approximated by Internet usage data, including search volume on Google and page view counts on Wikipedia [10]. We found strong correlations between search volume on Google and disease burden measured by prevalence and treatment cost for 39 diseases.

In this study, we extended our previous work by investigating millions of news articles to determine the coverage of various diseases and medical conditions as well as the sentiments and topics in news articles over the last 3 decades. The derived information could reflect the health problems and concerns of news media and the public in a similar way that social media have shown us [11-15]. In addition, news media influences our daily lives [16], has a vital role in politics, and affects public policy making [17]. Thus, our study could provide useful information on unmet medical needs and research priorities for the future and aid in decision making in public policy.

## Methods

### Workflow

**Figure 1:**
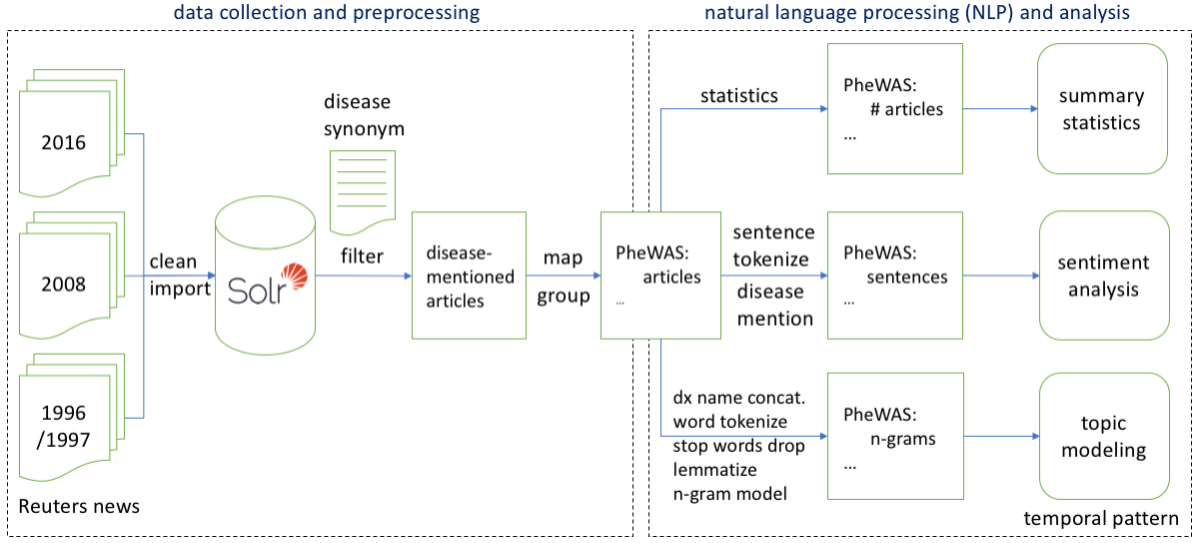
Workflow for mining news media data. Concat: concatenate; dx: disease; PheWAS: phenome-wide association study.

Our workflow to mine Reuters news data is illustrated in Figure 1 and consists of 2 main processing components: data collection and preprocessing, and natural language processing (NLP) and analysis. In the component of data collection and preprocessing, the 3 Reuters news corpora (Reuters Corpus Volume 1 [RCV1], Thomson Reuters Text Research Collection [TRC2], and Reuters Corpus 2016 [RC16]) were collected and cleaned due to heterogeneities and noise in the news articles. The cleaned data were then imported into Apache Solr (The Apache Software Foundation) for information indexing and searching. Solr is a popular, open-source, enterprise search platform built on Apache Lucene. The Reuters corpora were filtered in Solr to retrieve news articles that mentioned a list of disease synonyms from the Unified Medical Language System (UMLS) [18,19].

The UMLS assigns each disease concept a Concept Unique Identifier (CUI) and provides a disease synonym lookup table. It also provides the mapping between different controlled vocabularies, such as from the CUIs to the *International Classification of Diseases, Ninth Revision, Clinical Modification* (ICD-9-CM). Therefore we were able to group various names of the same disease concept to UMLS CUIs and then convert the CUIs to ICD-9-CM [20,21]. Then we aggregated different ICD-9-CM codes to phenome-wide association study (PheWAS) codes using their mapping table [22].

In NLP and analysis, we performed summary statistics, sentiment analysis, and topic modeling for the news articles grouped by PheWAS codes. To summarize the data, we calculated the percentage of news articles mentioning each PheWAS disease concept by dividing the number of articles mentioning each PheWAS concept by the total number of articles that mentioned all diseases and medical conditions in a year, that is 3516 in 1996/1997, 8835 in 2008, and 9633 in 2016. For sentiment analysis, articles that mentioned diseases and medical conditions were tokenized at the sentence level, and only sentences that mentioned diseases and medical conditions were selected to compute the sentiment scores. For topic modeling, all compound disease names in the news articles were replaced by their concatenated, single-word forms, in order to preserve the compound concepts as potential candidates of meaningful topics. For example, “lung cancer” was converted to “lung_cancer” before topic modeling. The news articles were then tokenized at the word level with stop words removed. A stop word is a commonly used word such as “the,” “and,” or “off” that is usually discarded in the text analysis. All words were lemmatized to reduce inflectional forms to a single base form. Finally, a n-gram (n=1-4) model was used to track meaningful short phrases in topic modeling. Sentiment analysis and topic modeling were performed using Valence Aware Dictionary and sEntiment Reasoner (VADER) in Natural Language Toolkit (NLTK) [23] and the Gensim library in Python [24].

Additional details about data collection, data cleaning, summary statistics, sentiment analysis, and topic modeling are discussed in the following sections.

### Data Collection

In this work, 3 news corpora in English were collected from the online newswire achieves of Reuters media (Reuters Group before 2008 and Thomson Reuters after 2008). Reuters media is a leading global information and news agency and the world’s largest international text and television news provider. The 3 Reuters news corpora used in this study, each in a span of a 1-year period, represent 3 decades (1990s, 2000s, and 2010s) and allow us to study the temporal trends of major topics in news media. Table 1 summarizes the dates and statistics of the 3 data sets including the numbers of all news articles, the numbers and percentages of articles that mentioned diseases and medical conditions, and the numbers of the mapped PheWAS codes [25].

RCV1 is the first official Reuters corpus [26,27] and is a large collection of over 800,000 news articles in English that were published from August 20, 1996, to August 19, 1997. In 2000, Reuters made this corpus available for free use by researchers. RCV1 has been extensively used for information retrieval and the development of NLP and machine learning tools [26].

TRC2 is another large newswire corpus of Reuters media. TRC2 includes more than 1,800,000 news articles produced by Reuters journalists from January 1, 2008, to February 28, 2009. This data set was initially provided to the participants of the 2009 blog track at the Text Retrieval Conference. In this study, we used the news articles from January 1, 2008, to December 31, 2008, which includes more than 1,500,000 news articles.

RCV1 and TRC2 are distributed free of charge by the National Institute of Standards and Technology and can be downloaded on request [28]. To analyze the latest news articles, we also created a third news article corpus from the Reuters archive websites [29-31] for 3 available English-speaking countries: United States, United Kingdom, and India.

**Table 1:** Summary statistics of Reuters historical news data.

Data set	Date	Article total, n	Articles mentioning diseases, n (%)	Mapped PheWAS^a^ codes
RCV1^b^	8/20/1996 to 8/19/1997	806,791	3516 (0.44)	342
TRC2^c^	1/1/2008 to 12/31/2008	1,546,350	8835 (0.57)	311
RC16^d^	1/1/2016 to 12/31/2016	1,182,761	9633 (0.81)	375

^a^PheWAS: phenome-wide association study.

^b^RCV1: Reuters Corpus Volume 1.

^c^TRC2: Thomson Reuters Text Research Collection.

^d^RC16: Reuters Corpus 2016.

The new Reuters news corpus is called RC16 for the purposes of this study. RC16 includes more than 1,100,000 news articles from January 1, 2016, to December 31, 2016.

### Data Cleaning

News articles we collected are mostly heterogeneous and noisy and need to be cleaned before text analysis. In practice, automatic data cleaning is relatively difficult and data cleaning needs to be performed on a case-by-case basis because heterogeneities and noises vary among raw text corpora [11]. For example, Reuters tags and editorial information exist in Reuters corpora, and some sentences are delimited by “>” or “*” in the news articles. These are different from tweet data used in our previous study on disease burden. We designed and used the following steps to clean the data retrieved from the 3 Reuters corpora.

- Removed Reuters tags such as “(Reuters)” and “(Reuters Life)”
- Removed editorial information. All sentences starting with “Editing by,” “Reporting by,” “Written by,” or “Page editor” were deleted
- Removed comments from news articles. Comments and remarks were discarded (eg, “click on the codes in brackets to see stories,” “the following statement was released by the rating agency,” and “note to subscribers: this top news page will change its name to healthcare from August 4 to better describe the sector it covers”)
- Replaced hyperlinks such as “http://topnews.session.rservices.com” with the word “link”
- Removed repeating special characters, for example, “---------------”
- Replaced the sentence delimiters “*” and “>” using a period. Replaced all uppercase letters with lowercase letters

The above steps cleaned up irrelevant metadata and special characters and are necessary for meaningful topic modeling and sentiment analysis. For example, the tag of Reuters appears at the end of each Reuters news article. If we do not remove it, the topic modeling results would be biased. In another case, the repeating special characters and sentence delimiters such as “*” and “>” can confuse the sentence segmentation process and impact the sentiment analysis results.

### Summary Statistics

We counted the total numbers of the news articles that mentioned all diseases and medical conditions in terms of a PheWAS disease concept. Due to different sizes of the 3 Reuters corpora, we adopted the coverage in percentage instead of the raw count of articles mentioning a PheWAS disease concept from 1 year to another. Therefore, we employed the normalized coverage (in percentage) to assess the coverage of different diseases and their temporal trends in the last 3 decades. In other word, we normalized the counts of disease-specific news articles by the total number of all news articles covering all diseases and medical conditions.

### Sentiment Analysis

Sentiment analysis refers to the computational treatment of subjectivity in texts to evaluate opinions, sentiments, attitudes, and emotions. It quantifies the sentiment contents in the given texts in a continuum scale (eg, [–1, 1]) [14,15].

We used the VADER module in NLTK for sentiment analysis. VADER is a lexicon- and rule-based sentiment analysis tool that is specifically optimized for sentiments expressed in social media. VADER reports a normalized and weighted compound score as a unidimensional measure of sentiment for a given sentence. The compound score of a sentence is computed by summing the predefined score of each word that exists in the sentiment lexicon. The compound score is then adjusted according to the embedded rules and normalized to be between −1.0 (the most negative) and 1.0 (the most positive), with 0.0 indicating neutral. The sentiment of each PheWAS disease concept was computed by averaging all sentence sentiments related to the PheWAS disease concept.

### Topic Modeling

Topic modeling automatically detects abstract topics in a collection of documents. Here, a topic refers to a repeating pattern of co-occurring terms in a text corpus. Intuitively, a set of words that appear most frequently represent the topic of a collection of documents. Topic modeling helps us to gain insights from large document collections by identifying hidden topics [11-13].

In this study, we used latent Dirichlet allocation (LDA), a state-of-art unsupervised topic-modeling technique [32]. LDA is a probabilistic model with a hierarchical structure for its components, which include documents, topics, and words. LDA assumes that a given document is generated from a mixture of topics and these topics produce the words in the documents according to their probabilistic distributions. LDA backtracks and derives the hidden topics that create those documents on the basis of the statistics of the included words.

More specifically, given a corpus of documents (D) with a vocabulary (V) and a preselected number of topics (K), the LDA model tries to infer 2 types of probability distributions, the probability of a topic *t* in a document *d*, P(t|d), and the probability of a word *w* associated with a topic *t*, P(w|t), where *d*, *t*, and *w* denote a document, a topic, and a word, respectively. The prior distributions of P(t|d) and P(w|t) are estimated from the Dirichlet distribution with 2 given hyperparameters, α and β. Because of the high number (K×D+V×K) of unknown probability densities, a Gibbs sample is applied to make iterative inference until these probability densities converge. In each inference iteration, P(t|d) is updated with the proportion of words currently assigned to a topic *t* in a document *d*, and P(w|t) is set to the proportion of a word linked to a topic *t* over all documents. Topic assignment of a word *w* in a document *d* is then updated with the topic *t* if the product of P(w|t)×P(t|d) achieves a maximal probability.

We used the LDA module in Gensim to identify the topics from the group of news articles that mentioned the same PheWAS disease concept. Gensim is a robust, open-source, topic modeling toolkit written in Python. Since we are mainly concerned with the specific contents of the disease-related topics and their temporal trends rather than the distribution of an arbitrary number of topics in each document, the number of topics in the LDA model is set to 1. The top 50 topic words were set to output, whereas all other parameters kept default values. Each topic model was iterated for at least 500 steps.

## Results

### Phenome-Wide Association Study Disease Concepts

After data preprocessing, 342 PheWAS disease concepts were found in RCV1, 311 PheWAS disease concepts in TRC2, and 375 PheWAS disease concepts in RC16, with 231 PheWAS disease concepts found in all 3 corpora. Summary statistics, sentiment analysis, and topic analysis were used to evaluate the news articles related to these 231 common PheWAS disease concepts. These results illustrate the temporal patterns that occurred in the last 3 decades.

### Summary Statistics

Table 1 summarizes the numbers and percentages of articles that mentioned diseases and medical conditions and the numbers of the mapped PheWAS disease concepts in the 3 Reuters corpora. The percentages of articles that mentioned diseases and medical conditions were 0.44% (3516/806,791), 0.57% (8835/1,546,350), and 0.81% (9633/1,182,761) in the 3 study periods, suggesting an increasing reporting coverage on diseases and medical conditions by Reuters media.

**Figure 2:**
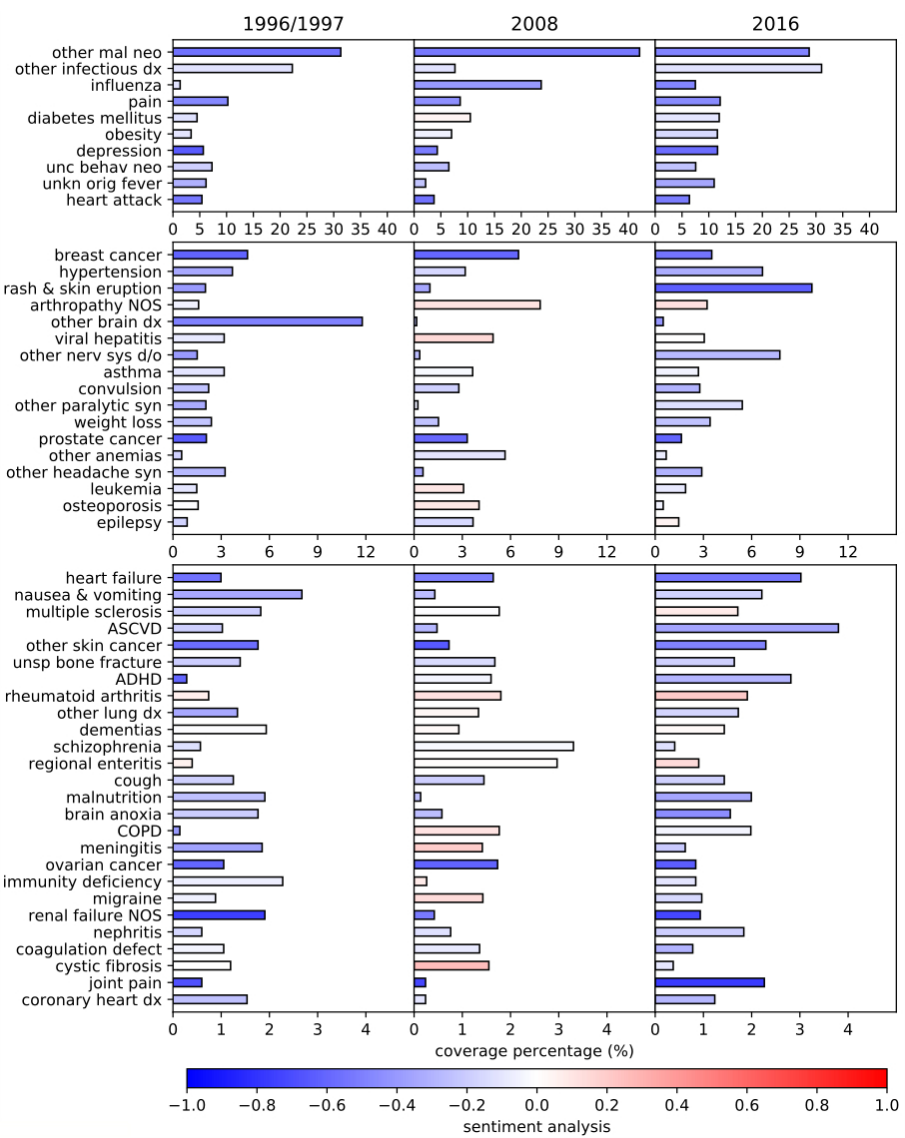
Coverage percentages and sentiments of the top 53 phenome-wide association study disease concepts. Blue, white, and red in a diverging color map denote the most negative (–1.0), right neutral (0.0), and the most positive (1.0) sentiments, respectively. The 53 phenome-wide association study disease concepts are put into 3 buckets based on coverage percentages for better resolution and comparison. From top to bottom, there are 10, 17, and 26 phenome-wide association study disease concepts in each budget with mean coverage range of 34% to 5%, 5% to 2%, and 2% to 1%, respectively. ADHD: attention-deficit/hyperactivity disorder; ASCVD: atherosclerotic cardiovascular disease; COPD: chronic obstructive pulmonary disease; dx: disease; mal neo: malignant neoplasm; NOS: not otherwise specified; other nerv sys d/o: other and unspecified disorders of the nervous system; unc behave: uncertain behaviour; unkn orig: unknown origin; unsp: unspecified; syn: syndrome.

We computed the coverage percentages of the 231 PheWAS disease concepts, and the results of coverage percentages can be found in Multimedia Appendix 1. Of the 231 PheWAS disease concepts, 53 have mean coverage percentages greater than 1% in the past 3 decades (1996/1997, 2008, and 2016), as shown in descending order in Figure 2. In total, 53 PheWAS disease concepts account for 90.27% (3174/3516) of the disease-mentioning news articles in RCV1, 94.76% (8372/8835) of the disease-mentioning news articles in TRC2, and 87.72% (8450/9633) of the disease-mentioning news articles in RC16, suggesting that these diseases were the major disease subjects reported by Reuters media. Some diseases, such as other malignant neoplasm, other infectious diseases, and influenza have dominating mean coverage percentages of 34%, 20%, and 11%, respectively, in the past 3 decades.

In addition, we analyzed the temporal trends of coverages of the 231 PheWAS disease concepts in the past 3 decades, and 4 major temporal trends were observed:

1. Some diseases maintained steady coverage percentages with small standard deviations in the last 3 decades. Examples include other malignant neoplasm (34.0% [SD 7.1%]), pain (10.3% [SD 1.8%]), neoplasm of uncertain behavior (7.1% [SD 0.6%]), asthma (3.2% [SD 0.5%]), convulsion (2.6% [SD 0.3%]), and multiple sclerosis (1.8% [SD 0.1%]).
2. Some diseases showed large fluctuations (ie, upward or downward swings) in their coverage percentages during the 3 decades. For instance, other infectious diseases, unknown original fever, other headache syndromes, other paralytic syndromes, and nausea and vomiting showed much lower coverage percentages in 2008 than in 1996/1997 and 2016. Some diseases such as arthropathy not otherwise specified, other anemias, leukemia, osteoporosis, epilepsy, and schizophrenia had very high coverage percentages in 2008 compared with 1996/1997 and 2016.
3. Steady increase refers to the overall growing of coverage percentages in the 3 study periods. For example, the coverage percentages of diabetes mellitus, obesity, heart failure, attention-deficit/hyperactivity disorder, and rheumatoid arthritis continually increased in the last 30 years. Influenza showed a much larger coverage percentage since 2008. The coverage percentages of depression, hypertension, and rash and skin eruption considerably increased in 2016.
4. Some diseases showed steady decreases: the coverage percentage of meningitis showed a continual decrease, for instance. The coverage percentage of other brain disease largely decreased since 2008. Cystic fibrosis showed a much lower coverage percentage in 2016 compared with 1996/1997 and 2008.

### Sentiment Analysis

Sentiment analysis was performed for all 231 PheWAS disease concepts, and the results were summarized in Multimedia Appendix 1. The sentiment scores of the 231 PheWAS disease concepts ranged from –0.94 to 0.73, with 226 of them having neutral or negative sentiments in news articles.

Figure 2 illustrates the sentiments of the 53 PheWAS disease concepts. While 43 diseases had neutral sentiments, the other 10 diseases, other malignant neoplasm, depression, heart attack, breast cancer, prostate cancer, heart failure, other skin cancer, ovarian cancer, renal failure not otherwise specified, and joint pain, had moderate negative sentiments with mean scores of approximately –0.59.

**Figure 3:**
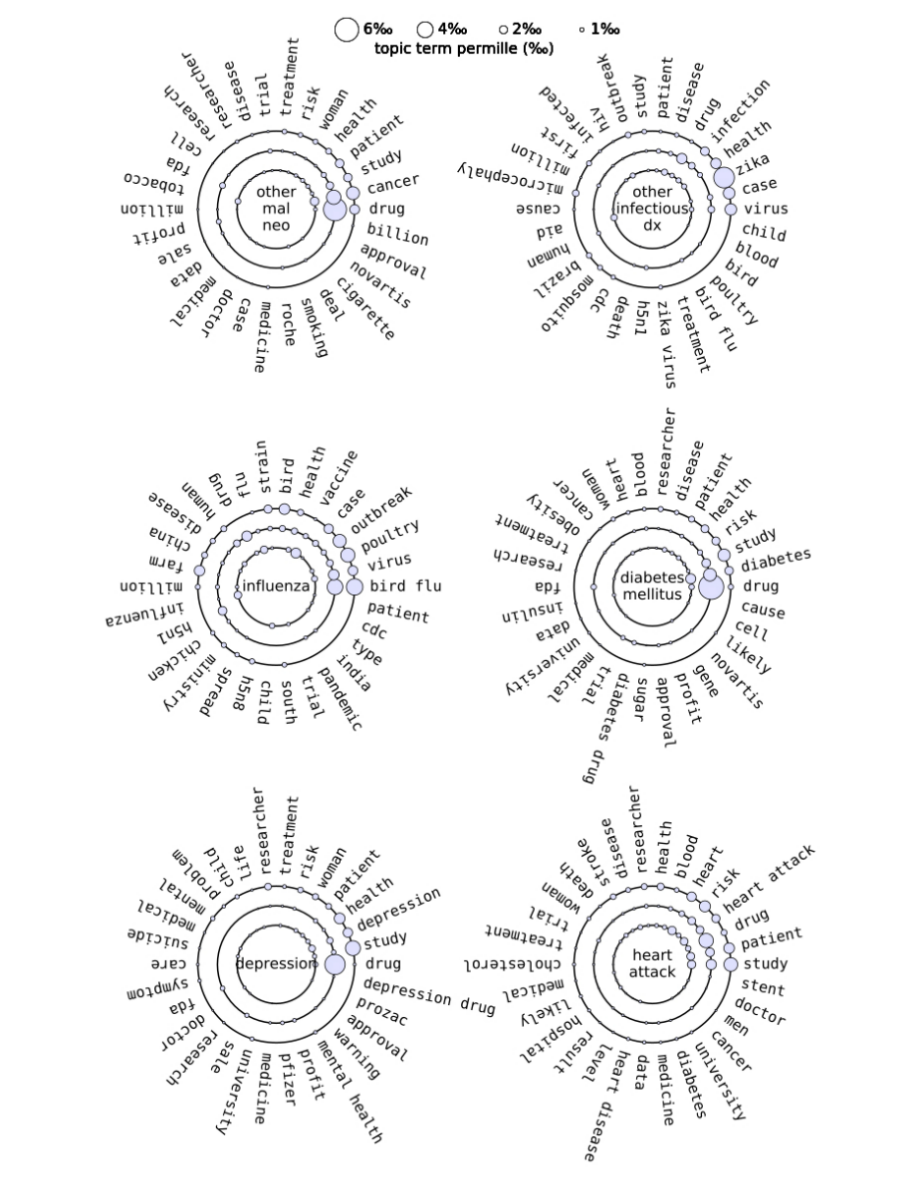
Top 30 topic keywords associated with the top 6 disease concepts in 3 decades. The rings represent the study periods of 1996/1997 (inner circle), 2008 (middle circle), and 2016 (outer circle). The size of the circles located on each ring denotes the permille of each term or phrase in a topic. dx: disease; mal neo: malignant neoplasm.

Interestingly, some diseases maintained steady sentiments from 1996 to 2016 (eg, other infectious diseases, pain, heart attack, prostate cancer, and ovarian cancer). A few diseases such as diabetes mellitus, depression, and hypertension showed fluctuations in sentiment. The sentiments of other malignant neoplasm and breast cancer gradually increased. Influenza had more negative sentiments in 2008 and 2016 compared with 1996/1997.

### Topic Modeling

Topic modeling results of the 231 PheWAS disease concepts are summarized in Multimedia Appendix 1. On the whole, the top 10 topic words occurring in the news articles about the 231 PheWAS disease concepts were drug, patient, health, study, risk, disease, treatment, researcher, trial, and FDA (US Food and Drug Administration). Figure 3 illustrates the topic contents of the 6 representative PheWAS disease concepts (other malignant neoplasm, other infectious diseases, influenza, diabetes mellitus, depression, and heart attack) in the 3 different study periods. The disease name for each PheWAS disease concept is shown in the center of the circle. The 30 most common topic words or phrases for each PheWAS disease concept are placed along small circles whose sizes represent the permille of each topic word or phrase in the topic content.

Two typical temporal patterns were found among the topics of the 231 PheWAS disease concepts in the past 3 decades.

1. There was no major change in the topic contents of some diseases over time or the permilles of key topic terms remained steady. Examples are diabetes mellitus, obesity, heart attack, breast cancer, and hypertension.
2. Some diseases showed considerable variation. Here considerable variation denotes that different or special terms arose in the topic contents of a certain disease in 1 or 2 study periods or the permilles of key topic words or phrases exhibited large variation. For example, the word smoking occurred in the topic contents of other malignant neoplasm only during 1996/1997. In the topic content of other infectious diseases, the permille of the word HIV has decreased by more than 50% since 2008.

## Discussion

### Combined Analysis

The top PheWAS disease concept was other malignant neoplasm. Its coverage remained steady at 34.0% in the last 3 decades (Figure 2). There are 4 other cancer-related diseases, breast cancer, prostate cancer, other skin cancer, and ovarian cancer, among the top 53 PheWAS disease concepts. Reuters media had a negative attitude toward all of these cancers. The large coverage percentages and negative sentiments indicate that Reuters media was strongly concerned with cancer, possibly because cancer is a primary global public health problem and is the second leading cause of death worldwide [33,34]. But Figure 2 shows that the overall sentiment scores have slightly increased since 1996. A 2017 report on cancer statistics noted that the overall cancer death rate declined by 25% from 1991 to 2014 in the United States as a result of a series of regulations and actions taken by the US government, such as tobacco control initiatives since the 1960s and cancer screenings [33]. Tobacco use is the most important risk factor for cancer and linked to about 80% to 90% of lung cancers [35]. Other malignant neoplasm was associated with the topic words tobacco, smoking, and cigarette in 1996/1997 but not in 2008 and 2016, which possibly reflects the progress in tobacco control in the United States (Table 2). Overall smoking prevalence decreased from 42.4% in 1965 to 16.8% in 2016, and the mortality rate due to lung cancer declined by 43% in men from 1990 to 2014 and by 17% in women from 2002 to 2014 [33]. In addition, breast cancer and prostate cancer are the top 2 and 3 cancers shown in Figure 2, which is consistent with a recent study that reported prostate cancer as the leading cancer in men and breast cancer as the leading cancer in women [33].

Other infectious diseases were the second most common PheWAS disease concept reported by Reuters media. Its coverage was 22.2% in 1996/1997, 7.6% in 2008, and 31.5% in 2016, which showed a large fluctuation in the last 30 years. Reuters media has neutral sentiment toward it, probably because infectious disease is no longer a major cause of death [36]. The topics associated with other infectious diseases varied with time. Two interesting terms that stand out in its topic content are HIV in 1996/1997 and Zika in 2016, suggesting that HIV and Zika virus probably attributed to the large fluctuation in news media coverage in the past 3 decades. The decrease in attention to HIV corresponded to a decline in the HIV/AIDS mortality rate after the introduction of antiretroviral therapy in 1995 [37]. Topics in 2016 included Zika-related words such as outbreak, microcephaly, Brazil, mosquito, and CDC (Centers for Disease Control and Prevention). The appearance of these topic words coincided with 2 events: the Zika virus outbreak in Brazil in 2015 (transmitted primarily by mosquitoes) and an association found at the same time between Zika virus infection and microcephaly [38,39].

The third most reported PheWAS disease concept was influenza. The coverage percentage of influenza dramatically increased from 1996 (1.3%) to 2008 (23.7%) and moderately decreased in 2016 (7.5%). The sentiment scores also largely declined from –0.11 (1996/1997) to –0.40 (2008) and slightly decreased to –0.48 (2016). In 2008 and 2016, the topic of influenza consisted of many terms related to avian influenza (eg, poultry, chicken, strain, H5N1, H5N8, outbreak, farm, China, India, and CDC). The outbreaks of avian influenza in 2008 and 2016 contributed to the high coverage percentages of influenza and received a large amount of attention from Reuters media [40-43]. The high mortality rate most likely resulted in the negative sentiment toward this disease [41].

**Table 2:** The permille of tobacco-related topic terms associated with other malignant neoplasm.

Topic term	1996/1997	2008	2016
Tobacco	1.5	0.0	0.0
Smoking	0.8	0.0	0.0
Cigarette	0.7	0.0	0.0

In addition to these top 3 covered PheWAS disease concepts, depression demonstrated some interesting findings. Its coverage percentage showed a 2-fold increase in 2016 compared with 1996/1997 and 2008, which is in accordance with a 2017 report published by the CDC [44]. The sentiment toward depression was negative in all 3 study periods, possibly because depression is the leading cause of disability worldwide and a major contributor to the overall global burden of disease [45]. Topics in news articles related to depression contained the words women, children, and suicide in 2016. The recent depression incidences have grown among adolescents and young adults, in particular among girls and young women [46], and suicide is often led by depression at its worst [47].

Here we discussed interesting observations and associations for 4 PheWAS disease concepts. However, further studies are needed to carefully and rigorously investigate the underlying causal events.

### Limitations

The study has some limitations. First, the data source from Reuters media in this study is limited to 3 sparse time periods: 1996/1997 (RCV1), 2008 (TRC2), and 2016 (RC16). These datasets do not include all the news articles from 1996 to 2016 and might create biases in the temporal trend analysis for the last 3 decades. However, fundamental biomedical changes do not occur in a day, a month, or a year for most diseases and medical conditions due to the slow development of the health care industry. The exception is infectious diseases, which come and go quickly.

Second, although Reuters media is a leading information and news agency and the largest international text and television news provider in the world, the news data used in this study might not be representative of all English-language news articles in the world. Reuters could have biases in their news report that were not taken into account in our analysis.

Third, we identified disease-mentioning news articles based on a disease synonym lookup table generated from UMLS. Although the table contains more than 20,000 disease synonyms for about 1800 PheWAS disease concepts, the disease synonym list may not guarantee all the mentions of disease and medical conditions in the Reuters news articles. Disease names may have a lot of morphologic variations and abbreviations. In addition, we converted disease synonyms to the PheWAS codes via a chain of mapping. In this process, the percentage of definitive mappings (one-to-one and multiple-to-one) from disease synonyms to PheWAS codes is 94%, which indicates that the upper bound of errors caused by ambiguous mappings (one-to-multiple or multiple-to-multiple mappings) might be 6%.

Fourth, the sentiment analysis and topic modeling step may also introduce bias. We used VADER for sentiment analysis in this study. It is a lexicon- and rule-based model. Although VADER has been used to analyze *New York Times* articles with a performance close to trained human raters, it has not been evaluated or tuned for Reuters news articles. On the other hand, LDA for topic modeling requires manual tuning parameters, including the number of topics, which can be more art than science. The perplexity plot shows the relationship between the perplexity and the topic numbers and thus provides limited hints on determining the optimal number of topics [48]. However, it still requires further topic visualization and input from domain experts to determine the optimal number of topics, which is not practical to do for 231 PheWAS disease concepts. We kept the number of topics to 1 in this study for all 231 PheWAS disease concepts, and other parameters were set to default values for practicability.

Finally, it would be valuable to perform correlation analysis between funding data and focuses in news articles to understand resource allocation. However, there is no comprehensive funding data for the large number of diseases and medical conditions.

### Conclusions

News media not only affects our daily lives but also plays a significant role in politics by influencing public policy making. In our study, we applied statistical analysis, sentiment analysis, and topic modeling techniques to over 3.5 million news articles from Reuters media in the past 3 decades in order to discover statistical and temporal patterns of coverage, sentiments, and topics for a large number of diseases and medical conditions. Our results show that Reuters media has gradually increased its coverage on diseases and medical conditions since 1996. Diseases and medical conditions like other malignant neoplasm, other infectious diseases, and influenza were the main focus of Reuters media with large coverage percentages, and 97.8% of diseases and medical conditions showed neutral or negative sentiments. Meaningful topics were identified in the news articles mentioning diseases and medical conditions. Combined analysis indicates statistical and temporal patterns of diseases and medical conditions that correspond to relevant policies and published research.

The infodemiological study expands our previous work on health resource allocation and disease burden estimation using online data. The multidimensional analysis of news media data enables the discoveries of disease-related focus, sentiments, and topics of news media. These discoveries could provide valuable information on unmet medical needs and research priorities and offer guidance for future decision making in public policy.

## Appendix

Multimedia Appendix 1 Coverage percentages, topics, and sentiments of 231 phenome-wide association study disease concepts.

## Abbreviations

CDC: Centers for Disease Control and Prevention
CUI: Concept Unique Identifier
DALY: disability-adjusted life years
FDA: US Food and Drug Administration
ICD-9-CM: International Classification of Diseases, Ninth Revision, Clinical Modification
LDA: latent Dirichlet allocation
NIH: National Institutes of Health
NLP: natural language processing
NLTK: Natural Language Toolkit
PheWAS: phenome-wide association study
PHI: Public Health Index
QALY: quality-adjusted life years
RC16: Reuters Corpus 2016
RCV1: Reuters Corpus Volume 1
ROI: Research Opportunity Index
TRC2: Thomson Reuters Text Research Collection
UMLS: Unified Medical Language System
VADER: Valence Aware Dictionary and sEntiment Reasoner
